# *catena*-Poly[dieth­yl(2-hydroxy­ethyl)­ammonium [[tetra-μ-acetato-κ^8^ *O*:*O*′-dicuprate(II)(*Cu*—*Cu*)]-μ-acetato-κ^2^ *O*:*O*′] dichloro­methane solvate]

**DOI:** 10.1107/S1600536808044048

**Published:** 2009-01-08

**Authors:** Muhammad Shahid, Muhammad Mazhar, Paul O’Brien, Mohammad Afzaal, James Raftery

**Affiliations:** a Department of Chemistry, Quaid-i-Azam University, Islamabad 45320, Pakistan; b The School of Chemistry, The University of Manchester, Oxford Road, Manchester M13 9PL, England

## Abstract

The title compound, {(C_6_H_16_NO)[Cu_2_(CH_3_COO)_5_]·CH_2_Cl_2_}_*n*_, consists of acetate-bridged Cu_2_(CH_3_COO)_4_ units that are connected *via* another acetate anion at each terminus to form infinite anionic [{Cu_2_(CH_3_COO)_4_}(CH_3_COO)]_*n*_ chains along [100]. The connecting acetate is hydrogen bonded to the dieth­yl(2-hydroxy­ethyl)ammonium cation, and the dichloro­methane solvent mol­ecule fills the remaining voids in the structure. The O—Cu—Cu angles along the polymeric chain are nearly linear [175.49 (5)°], but individual O—Cu—Cu—O units along the chain are bent and rotated against each other at the bridging acetate ion. Translation of each Cu_2_(CH_3_COO)_4_ unit along the chain, represented by the least-squares plane of the two copper ions along with four of the acetate O atoms, rotated these units by 35.16 (3)°.

## Related literature

Shahid, Mazhar, Helliwell *et al.* (2008 ▶) describe the study of dinuclear Cu complexes; Van Niekerk & Schoening (1953 ▶) provide X-ray evidence for Cu—Cu bonds in cupric acetate; Brown & Chidambaram (1973 ▶) report the redetermination of the structure of cupric acetate by neutron-diffraction; Shahid, Mazhar, Malik *et al.* (2008 ▶); Hamid *et al.* (2007 ▶) and Zhang *et al.* (2004 ▶) describe geometric parameters of organo–copper complexes. 
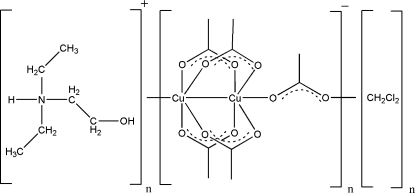


## Experimental

### 

#### Crystal data

(C_6_H_16_NO)[Cu_2_(C_2_H_3_O_2_)_5_]·CH_2_Cl_2_
*M* *_r_* = 625.42
Orthorhombic, 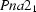

*a* = 17.6366 (11) Å
*b* = 12.1078 (8) Å
*c* = 11.9148 (7) Å
*V* = 2544.3 (3) Å^3^
*Z* = 4
Mo *K*α radiation
μ = 1.94 mm^−1^
*T* = 100 (2) K
0.40 × 0.40 × 0.10 mm

#### Data collection

Bruker SMART CCD area-detector diffractometer
Absorption correction: multi-scan (*SADABS*; Bruker, 2001 ▶) *T*_min_ = 0.657, *T*_max_ = 0.830
21202 measured reflections
5939 independent reflections
5693 reflections with *I* > 2σ(*I*)
*R*_int_ = 0.029

#### Refinement

*R*[*F* ^2^ > 2σ(*F* ^2^)] = 0.034
*wR*(*F* ^2^) = 0.080
*S* = 1.08
5939 reflections
306 parameters
1 restraint
H-atom parameters constrained
Δρ_max_ = 0.75 e Å^−3^
Δρ_min_ = −0.40 e Å^−3^
Absolute structure: Flack (1983 ▶), 2726 Friedel pairs
Flack parameter: 0.017 (11)

### 

Data collection: *SMART* (Bruker, 2001 ▶); cell refinement: *SAINT-Plus* (Bruker, 2003 ▶); data reduction: *SAINT-Plus*; program(s) used to solve structure: *SHELXS97* (Sheldrick, 2008 ▶); program(s) used to refine structure: *SHELXL97* (Sheldrick, 2008 ▶); molecular graphics: *SHELXTL* (Sheldrick, 2008 ▶); software used to prepare material for publication: *SHELXTL*.

## Supplementary Material

Crystal structure: contains datablocks I, global. DOI: 10.1107/S1600536808044048/zl2161sup1.cif

Structure factors: contains datablocks I. DOI: 10.1107/S1600536808044048/zl2161Isup2.hkl

Additional supplementary materials:  crystallographic information; 3D view; checkCIF report

## Figures and Tables

**Table 1:** Hydrogen-bond geometry (Å, °)

*D*—H⋯*A*	*D*—H	H⋯*A*	*D*⋯*A*	*D*—H⋯*A*
N1—H1⋯O10^i^	0.93	1.97	2.832 (4)	153
N1—H1⋯O9^i^	0.93	2.45	3.056 (3)	123
O11—H11⋯O9^i^	0.84	2.04	2.840 (3)	159

Symmetry code: (i) 
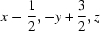
.

**Fig. 1.**
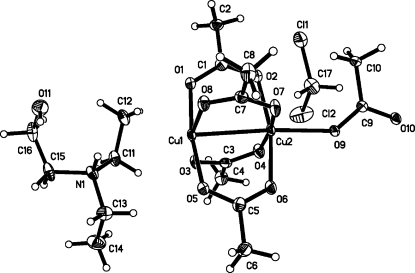
View of the title compound (50% probability displacement ellipsoids)

**Fig. 2.**
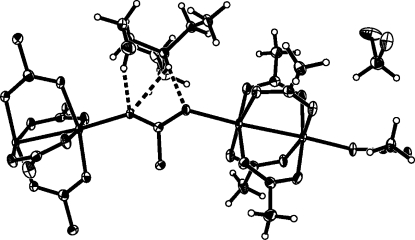
Fragment of the chain showing the H-bonding interactions.

**Fig. 3.**
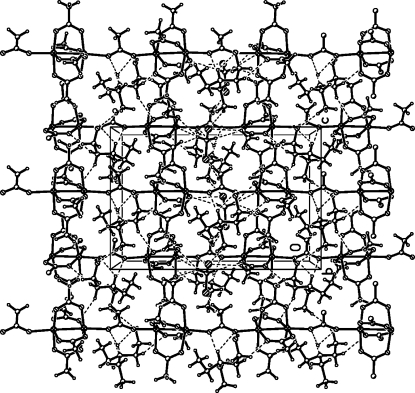
View down the b axis showing the infinite chains.

## supplementary crystallographic information

### Comment

The background of this study has been set out in our previous work on the structural chemistry of metal-organic compounds (Shahid, Mazhar, Helliwell *et al.*, 2008). Herein, as a continuation of these studies, the structure of the title compound is described which consists of acetate bridged Cu_2_(CH_3_COO)_4_ units that are connected *via* another acetate anion at each terminus to form infinite anionic [{Cu_2_(CH_3_COO)_4_}(CH_3_COO)]_n_ chains along the [100] direction of the crystal. Crystallographically speaking the chain is generated from *a* glide related copies of the monomer. The connecting acetate is hydrogen bonded to the (diethylammonium)ethanol cation (Fig. 2). The dichloromethane solvate molecule occupies voids in the structure. The O—Cu—Cu angles along the polymeric chain are nearly linear (175.49 (5)°), but individual O—Cu—Cu—O units along the chain are rotated relative to each other. Representing the orientation of Cu_2_(CH_3_COO)_4_ unit by the least squares plane Cu1 Cu2 O1 O2 O5 O6, translation along the chain rotates the orientation by 35.16 (3)°.

In the title compound (Fig.1), the two metal centers are similar; each has a coordination number of six having a coordination geometry close to octahedral, with a CuO_5_Cu core similar to that of Cu centers in Cu_2_(OAc)_4_(H_2_O)_2_. The basal planes of Cu(1) and Cu(2) are each composed of an oxygen from each of the four acetate groups (O(1), O(3), O(5), O(8) and O(2), O(4), O(6), O(7) respectively), which link the two copper atoms in the monomer. Coordination by the fifth acetate's O atoms, O(9) and O(10) (from a symmetry generated copy), form one apical bond for Cu(2) and Cu(1) respectively. The octahedral coordination of the copper atoms is completed by the apical Cu(1)—Cu(2) bond of 2.6259 (4) Å. This is significantly shorter than the 2.64 Å as reported for dinuclear copper (II) acetate monohydrate in 1953 (Van Niekerk & Schoening, 1953), but close to the more accurate value obtained in a redetermination by neutron diffraction analysis (2.6143 (17) Å, Brown & Chidambaram, 1973). The Cu—O bond lengths in the basal planes for both the Cu atoms range from 1.949 (2) to 1.985 (2) Å and the average distance is in good agreement with 1.97 Å, as reported for copper acetate (Van Niekerk & Schoening, 1953). The most striking structural difference between the title compound and the dinuclear units in cupric acetate appears to be the weaker apixal bonds Cu—O which are 2.148 (18) and 2.124 (18) Å for Cu(1) and Cu(2), respectively in the title compound and 2.20 Å in the cupric acetate. The distortion is further evident from the slight deviation of *trans* angles in the basal plane and axial angle from ideal value of 180°. This is in good agreement with the literature (Shahid, Mazhar, Malik *et al.*, 2008); Hamid *et al.*, 2007; Zhang *et al.*, 2004). In the structure, the (diethylamonium)ethanol cations are linked through hydrogen bonds [O(11)—H(11)···O(9)], [N(1)—H(1)···O(9)] and [N(1)—H(1)···O(10)] to the connecting acetate group occupying *cis* positions at the main polymeric chain (Table 1, Fig. 3).

### Experimental

*N*,*N*-Diethylaminoethanol (deaeH) (0.27 g, 2.34 mmol) and acetic acid (0.14 g, 2.34 mmol) were added to a stirred suspension of Cu(CH_3_COO)_2_.H_2_O (0.85 g, 4.67 mmol) in 25 ml dichloromethane. After two hours stirring, the mixture was vacuum evaporated to dryness and the solid was redissolved in minimum amount of dichloromethane to give blue block-shaped crystals at room temperature after two weeks.

### Refinement

The non-hydrogen atoms were refined anisotropically. H atoms were included in calculated positions with C—H lengths of 0.95(CH), 0.99(CH_2_) & 0.98(CH_3_)Å; *U*_iso_(H) values were fixed at 1.2*U*_eq_(C) except for CH_3_ where it was 1.5*U*_eq_(C). For N—H and O—H the lengths and *U*_iso_ were 0.98Å and 1.2*U*_eq_(N) and 0.84Å and 1.5*U*_eq_(O) respectively.

### Crystal data

**Table:**

(C_6_H_16_NO)[Cu_2_(C_2_H_3_O_2_)_5_]·CH_2_Cl_2_	*D*_x_ = 1.633 Mg m^−^^3^
*M**_r_* = 625.42	Mo *K*α radiation, λ = 0.71073 Å
Orthorhombic, *P**n**a*2_1_	Cell parameters from 7814 reflections
*a* = 17.6366 (11) Å	θ = 2.4–28.1°
*b* = 12.1078 (8) Å	µ = 1.94 mm^−^^1^
*c* = 11.9148 (7) Å	*T* = 100 K
*V* = 2544.3 (3) Å^3^	Plate, turquoise
*Z* = 4	0.40 × 0.40 × 0.10 mm
*F*(000) = 1288	

### Data collection

**Table:**

Bruker SMART CCD area-detector diffractometer	5939 independent reflections
Radiation source: fine-focus sealed tube	5693 reflections with *I* > 2σ(*I*)
graphite	*R*_int_ = 0.029
φ and ω scans	θ_max_ = 28.3°, θ_min_ = 2.0°
Absorption correction: multi-scan (*SADABS*; Bruker, 2001)	*h* = −22→22
*T*_min_ = 0.657, *T*_max_ = 0.830	*k* = −16→15
21202 measured reflections	*l* = −15→15

### Refinement

**Table:**

Refinement on *F*^2^	Secondary atom site location: difference Fourier map
Least-squares matrix: full	Hydrogen site location: inferred from neighbouring sites
*R*[*F*^2^ > 2σ(*F*^2^)] = 0.034	H-atom parameters constrained
*wR*(*F*^2^) = 0.080	*w* = 1/[σ^2^(*F*_o_^2^) + (0.0393*P*)^2^ + 1.2652*P*] where *P* = (*F*_o_^2^ + 2*F*_c_^2^)/3
*S* = 1.08	(Δ/σ)_max_ = 0.013
5939 reflections	Δρ_max_ = 0.75 e Å^−^^3^
306 parameters	Δρ_min_ = −0.40 e Å^−^^3^
1 restraint	Absolute structure: Flack (1983), 2726 Friedel pairs
Primary atom site location: structure-invariant direct methods	Flack parameter: 0.017 (11)

### Special details

**Table:**

Geometry. All e.s.d.'s (except the e.s.d. in the dihedral angle between two l.s. planes) are estimated using the full covariance matrix. The cell e.s.d.'s are taken into account individually in the estimation of e.s.d.'s in distances, angles and torsion angles; correlations between e.s.d.'s in cell parameters are only used when they are defined by crystal symmetry. An approximate (isotropic) treatment of cell e.s.d.'s is used for estimating e.s.d.'s involving l.s. planes.
Refinement. Refinement of *F*^2^ against ALL reflections. The weighted *R*-factor *wR* and goodness of fit *S* are based on *F*^2^, conventional *R*-factors *R* are based on *F*, with *F* set to zero for negative *F*^2^. The threshold expression of *F*^2^ > σ(*F*^2^) is used only for calculating *R*-factors(gt) *etc*. and is not relevant to the choice of reflections for refinement. *R*-factors based on *F*^2^ are statistically about twice as large as those based on *F*, and *R*- factors based on ALL data will be even larger.

### Fractional atomic coordinates and isotropic or equivalent isotropic displacement parameters (Å^2^)

**Table:**

	*x*	*y*	*z*	*U*_iso_*/*U*_eq_	
O10	1.03029 (10)	0.66475 (16)	0.5408 (2)	0.0152 (4)	
C1	0.75148 (17)	0.9544 (3)	0.6165 (2)	0.0176 (6)	
C2	0.77083 (18)	1.0672 (3)	0.6609 (3)	0.0206 (6)	
H2A	0.7588	1.0707	0.7411	0.031*	
H2B	0.7413	1.1230	0.6206	0.031*	
H2C	0.8250	1.0813	0.6500	0.031*	
C3	0.73249 (17)	0.8234 (2)	0.3482 (2)	0.0166 (6)	
C4	0.73934 (19)	0.8662 (3)	0.2292 (3)	0.0222 (7)	
H4A	0.7896	0.8478	0.1994	0.033*	
H4B	0.7327	0.9465	0.2289	0.033*	
H4C	0.7002	0.8319	0.1823	0.033*	
C5	0.68347 (17)	0.5689 (3)	0.4849 (3)	0.0191 (6)	
C6	0.66262 (19)	0.4540 (3)	0.4463 (3)	0.0283 (8)	
H6A	0.7085	0.4087	0.4414	0.042*	
H6B	0.6384	0.4581	0.3724	0.042*	
H6C	0.6274	0.4208	0.5002	0.042*	
C7	0.71363 (17)	0.6985 (3)	0.7522 (3)	0.0173 (6)	
C8	0.71030 (19)	0.6623 (3)	0.8732 (3)	0.0257 (7)	
H8A	0.6693	0.7017	0.9116	0.039*	
H8B	0.7587	0.6791	0.9100	0.039*	
H8C	0.7008	0.5826	0.8767	0.039*	
C9	0.96959 (15)	0.7062 (2)	0.5798 (2)	0.0133 (6)	
C10	0.97463 (17)	0.7894 (3)	0.6744 (3)	0.0189 (6)	
H10A	1.0156	0.7682	0.7256	0.028*	
H10B	0.9265	0.7909	0.7154	0.028*	
H10C	0.9851	0.8628	0.6433	0.028*	
C11	0.54065 (17)	0.9990 (3)	0.2860 (3)	0.0210 (7)	
H11A	0.5795	0.9515	0.2504	0.025*	
H11B	0.5183	1.0463	0.2268	0.025*	
C12	0.57806 (18)	1.0711 (3)	0.3723 (3)	0.0240 (7)	
H12A	0.5967	1.0253	0.4342	0.036*	
H12B	0.5412	1.1246	0.4012	0.036*	
H12C	0.6206	1.1106	0.3379	0.036*	
C13	0.4783 (2)	0.8130 (3)	0.2850 (3)	0.0265 (7)	
H13A	0.4413	0.7669	0.3262	0.032*	
H13B	0.5290	0.7792	0.2947	0.032*	
C14	0.4581 (2)	0.8124 (4)	0.1617 (3)	0.0350 (9)	
H14A	0.4934	0.8599	0.1205	0.052*	
H14B	0.4063	0.8399	0.1519	0.052*	
H14C	0.4615	0.7368	0.1327	0.052*	
C15	0.40137 (17)	0.9772 (3)	0.3296 (3)	0.0249 (7)	
H15A	0.3913	0.9998	0.2512	0.030*	
H15B	0.3635	0.9203	0.3499	0.030*	
C16	0.39024 (18)	1.0753 (3)	0.4043 (3)	0.0280 (7)	
H16A	0.4225	1.1365	0.3771	0.034*	
H16B	0.3368	1.0996	0.3991	0.034*	
C17	0.9547 (2)	0.9492 (3)	0.4356 (3)	0.0292 (8)	
H17A	0.9991	0.9018	0.4520	0.035*	
H17B	0.9086	0.9093	0.4601	0.035*	
Cl1	0.96272 (5)	1.07456 (9)	0.51185 (9)	0.0367 (2)	
Cl2	0.94975 (7)	0.97421 (9)	0.29049 (9)	0.0475 (3)	
Cu1	0.647405 (16)	0.78765 (2)	0.55145 (3)	0.01289 (8)	
Cu2	0.791704 (16)	0.73425 (3)	0.54933 (4)	0.01346 (8)	
N1	0.47907 (14)	0.9266 (2)	0.3355 (2)	0.0182 (5)	
H1	0.4905	0.9179	0.4112	0.022*	
O1	0.68332 (11)	0.93608 (17)	0.59168 (18)	0.0168 (4)	
O2	0.80508 (12)	0.88517 (18)	0.60953 (19)	0.0211 (5)	
O3	0.66724 (12)	0.82111 (19)	0.39116 (18)	0.0184 (4)	
O4	0.79321 (12)	0.79520 (19)	0.39662 (19)	0.0194 (5)	
O5	0.62993 (11)	0.63224 (17)	0.51139 (19)	0.0193 (4)	
O6	0.75254 (11)	0.59322 (18)	0.4873 (2)	0.0202 (5)	
O7	0.77446 (12)	0.67809 (19)	0.70047 (19)	0.0212 (5)	
O8	0.65654 (12)	0.7454 (2)	0.71140 (18)	0.0188 (4)	
O9	0.90593 (10)	0.67911 (16)	0.5416 (2)	0.0172 (4)	
O11	0.40773 (14)	1.05419 (19)	0.5170 (2)	0.0306 (6)	
H11	0.3963	0.9886	0.5327	0.046*	

### Atomic displacement parameters (Å^2^)

**Table:**

	*U*^11^	*U*^22^	*U*^33^	*U*^12^	*U*^13^	*U*^23^
O10	0.0095 (8)	0.0212 (9)	0.0148 (10)	−0.0006 (6)	0.0011 (8)	−0.0015 (10)
C1	0.0178 (15)	0.0243 (16)	0.0107 (14)	−0.0011 (12)	0.0035 (11)	0.0009 (12)
C2	0.0197 (15)	0.0206 (16)	0.0215 (15)	−0.0048 (12)	0.0025 (13)	−0.0057 (12)
C3	0.0190 (14)	0.0160 (14)	0.0148 (14)	−0.0009 (11)	−0.0020 (12)	−0.0026 (11)
C4	0.0220 (16)	0.0293 (17)	0.0153 (15)	0.0007 (13)	0.0025 (12)	0.0042 (13)
C5	0.0186 (15)	0.0205 (15)	0.0182 (15)	0.0010 (12)	0.0025 (12)	0.0014 (12)
C6	0.0172 (16)	0.0200 (16)	0.048 (2)	−0.0038 (12)	0.0046 (15)	−0.0101 (15)
C7	0.0172 (15)	0.0179 (14)	0.0168 (15)	−0.0019 (11)	0.0000 (11)	−0.0016 (12)
C8	0.0232 (16)	0.0366 (19)	0.0173 (16)	0.0060 (14)	0.0024 (13)	0.0091 (14)
C9	0.0139 (13)	0.0139 (13)	0.0123 (15)	−0.0011 (10)	−0.0003 (10)	0.0017 (9)
C10	0.0144 (14)	0.0209 (16)	0.0213 (16)	0.0000 (11)	−0.0036 (12)	−0.0091 (12)
C11	0.0150 (15)	0.0296 (18)	0.0185 (16)	−0.0010 (12)	0.0033 (12)	0.0075 (13)
C12	0.0144 (15)	0.0294 (17)	0.0283 (17)	−0.0048 (13)	−0.0006 (13)	0.0063 (14)
C13	0.0315 (19)	0.0241 (16)	0.0240 (17)	−0.0013 (14)	−0.0016 (14)	0.0000 (14)
C14	0.040 (2)	0.043 (2)	0.0215 (17)	−0.0088 (17)	−0.0011 (16)	−0.0045 (16)
C15	0.0135 (15)	0.0404 (19)	0.0208 (16)	−0.0001 (13)	−0.0038 (12)	0.0141 (14)
C16	0.0133 (15)	0.0298 (17)	0.041 (2)	0.0028 (13)	0.0025 (14)	0.0116 (15)
C17	0.0277 (18)	0.036 (2)	0.0242 (18)	−0.0045 (14)	0.0010 (15)	0.0061 (15)
Cl1	0.0248 (4)	0.0460 (5)	0.0393 (5)	0.0034 (4)	−0.0039 (4)	−0.0083 (4)
Cl2	0.0826 (8)	0.0327 (5)	0.0272 (5)	−0.0082 (5)	0.0083 (5)	0.0062 (4)
Cu1	0.00720 (13)	0.01831 (15)	0.01316 (15)	0.00109 (10)	0.00003 (17)	−0.00089 (17)
Cu2	0.00722 (13)	0.01905 (15)	0.01411 (15)	0.00129 (10)	0.00009 (19)	−0.00129 (18)
N1	0.0148 (12)	0.0255 (14)	0.0142 (12)	−0.0033 (10)	−0.0022 (10)	0.0056 (11)
O1	0.0117 (10)	0.0184 (10)	0.0205 (10)	0.0008 (8)	−0.0007 (8)	−0.0027 (8)
O2	0.0119 (10)	0.0253 (12)	0.0260 (12)	0.0025 (9)	−0.0020 (9)	−0.0077 (10)
O3	0.0117 (10)	0.0290 (12)	0.0145 (10)	0.0008 (9)	−0.0004 (8)	0.0014 (9)
O4	0.0127 (10)	0.0295 (12)	0.0161 (11)	0.0003 (8)	0.0025 (8)	0.0018 (9)
O5	0.0123 (10)	0.0200 (10)	0.0255 (11)	−0.0005 (8)	−0.0006 (8)	−0.0045 (8)
O6	0.0103 (10)	0.0207 (11)	0.0295 (13)	−0.0016 (8)	0.0002 (9)	−0.0022 (9)
O7	0.0138 (10)	0.0317 (12)	0.0183 (11)	0.0069 (9)	0.0001 (9)	0.0054 (9)
O8	0.0153 (10)	0.0273 (12)	0.0137 (10)	0.0043 (9)	0.0023 (8)	0.0023 (9)
O9	0.0083 (8)	0.0220 (9)	0.0213 (11)	0.0002 (7)	−0.0008 (10)	−0.0075 (10)
O11	0.0310 (13)	0.0231 (11)	0.0378 (15)	−0.0031 (10)	0.0034 (11)	−0.0001 (10)

### Geometric parameters (Å, °)

**Table:**

O10—C9	1.271 (3)	C12—H12A	0.9800
O10—Cu1^i^	2.1482 (18)	C12—H12B	0.9800
C1—O1	1.258 (4)	C12—H12C	0.9800
C1—O2	1.266 (4)	C13—N1	1.500 (4)
C1—C2	1.504 (4)	C13—C14	1.512 (5)
C2—H2A	0.9800	C13—H13A	0.9900
C2—H2B	0.9800	C13—H13B	0.9900
C2—H2C	0.9800	C14—H14A	0.9800
C3—O3	1.260 (4)	C14—H14B	0.9800
C3—O4	1.263 (4)	C14—H14C	0.9800
C3—C4	1.514 (4)	C15—C16	1.496 (5)
C4—H4A	0.9800	C15—N1	1.503 (4)
C4—H4B	0.9800	C15—H15A	0.9900
C4—H4C	0.9800	C15—H15B	0.9900
C5—O6	1.254 (4)	C16—O11	1.400 (4)
C5—O5	1.257 (4)	C16—H16A	0.9900
C5—C6	1.510 (4)	C16—H16B	0.9900
C6—H6A	0.9800	C17—Cl2	1.758 (4)
C6—H6B	0.9800	C17—Cl1	1.775 (4)
C6—H6C	0.9800	C17—H17A	0.9900
C7—O8	1.254 (4)	C17—H17B	0.9900
C7—O7	1.262 (4)	Cu1—O1	1.965 (2)
C7—C8	1.508 (4)	Cu1—O5	1.966 (2)
C8—H8A	0.9800	Cu1—O8	1.980 (2)
C8—H8B	0.9800	Cu1—O3	1.983 (2)
C8—H8C	0.9800	Cu1—O10^ii^	2.1482 (18)
C9—O9	1.255 (3)	Cu1—Cu2	2.6259 (4)
C9—C10	1.514 (4)	Cu2—O7	1.949 (2)
C10—H10A	0.9800	Cu2—O4	1.964 (2)
C10—H10B	0.9800	Cu2—O2	1.977 (2)
C10—H10C	0.9800	Cu2—O6	1.985 (2)
C11—C12	1.502 (5)	Cu2—O9	2.1243 (18)
C11—N1	1.515 (4)	N1—H1	0.9300
C11—H11A	0.9900	O11—H11	0.8400
C11—H11B	0.9900		
			
C9—O10—Cu1^i^	133.03 (19)	C13—C14—H14C	109.5
O1—C1—O2	125.5 (3)	H14A—C14—H14C	109.5
O1—C1—C2	117.4 (3)	H14B—C14—H14C	109.5
O2—C1—C2	117.1 (3)	C16—C15—N1	114.6 (3)
C1—C2—H2A	109.5	C16—C15—H15A	108.6
C1—C2—H2B	109.5	N1—C15—H15A	108.6
H2A—C2—H2B	109.5	C16—C15—H15B	108.6
C1—C2—H2C	109.5	N1—C15—H15B	108.6
H2A—C2—H2C	109.5	H15A—C15—H15B	107.6
H2B—C2—H2C	109.5	O11—C16—C15	113.4 (3)
O3—C3—O4	125.6 (3)	O11—C16—H16A	108.9
O3—C3—C4	117.4 (3)	C15—C16—H16A	108.9
O4—C3—C4	116.9 (3)	O11—C16—H16B	108.9
C3—C4—H4A	109.5	C15—C16—H16B	108.9
C3—C4—H4B	109.5	H16A—C16—H16B	107.7
H4A—C4—H4B	109.5	Cl2—C17—Cl1	111.1 (2)
C3—C4—H4C	109.5	Cl2—C17—H17A	109.4
H4A—C4—H4C	109.5	Cl1—C17—H17A	109.4
H4B—C4—H4C	109.5	Cl2—C17—H17B	109.4
O6—C5—O5	125.5 (3)	Cl1—C17—H17B	109.4
O6—C5—C6	117.4 (3)	H17A—C17—H17B	108.0
O5—C5—C6	117.1 (3)	O1—Cu1—O5	170.19 (8)
C5—C6—H6A	109.5	O1—Cu1—O8	88.59 (10)
C5—C6—H6B	109.5	O5—Cu1—O8	89.95 (10)
H6A—C6—H6B	109.5	O1—Cu1—O3	89.50 (9)
C5—C6—H6C	109.5	O5—Cu1—O3	89.39 (10)
H6A—C6—H6C	109.5	O8—Cu1—O3	164.87 (9)
H6B—C6—H6C	109.5	O1—Cu1—O10^ii^	94.53 (8)
O8—C7—O7	125.6 (3)	O5—Cu1—O10^ii^	95.26 (8)
O8—C7—C8	118.1 (3)	O8—Cu1—O10^ii^	101.81 (9)
O7—C7—C8	116.3 (3)	O3—Cu1—O10^ii^	93.31 (9)
C7—C8—H8A	109.5	O1—Cu1—Cu2	85.13 (6)
C7—C8—H8B	109.5	O5—Cu1—Cu2	85.06 (6)
H8A—C8—H8B	109.5	O8—Cu1—Cu2	82.34 (6)
C7—C8—H8C	109.5	O3—Cu1—Cu2	82.54 (6)
H8A—C8—H8C	109.5	O10^ii^—Cu1—Cu2	175.83 (7)
H8B—C8—H8C	109.5	O7—Cu2—O4	171.68 (9)
O9—C9—O10	121.2 (3)	O7—Cu2—O2	90.33 (10)
O9—C9—C10	119.7 (2)	O4—Cu2—O2	89.27 (10)
O10—C9—C10	119.1 (2)	O7—Cu2—O6	89.42 (10)
C9—C10—H10A	109.5	O4—Cu2—O6	89.01 (10)
C9—C10—H10B	109.5	O2—Cu2—O6	166.35 (9)
H10A—C10—H10B	109.5	O7—Cu2—O9	94.49 (9)
C9—C10—H10C	109.5	O4—Cu2—O9	93.75 (9)
H10A—C10—H10C	109.5	O2—Cu2—O9	101.13 (8)
H10B—C10—H10C	109.5	O6—Cu2—O9	92.50 (8)
C12—C11—N1	112.6 (3)	O7—Cu2—Cu1	85.74 (6)
C12—C11—H11A	109.1	O4—Cu2—Cu1	85.95 (6)
N1—C11—H11A	109.1	O2—Cu2—Cu1	83.37 (6)
C12—C11—H11B	109.1	O6—Cu2—Cu1	83.00 (6)
N1—C11—H11B	109.1	O9—Cu2—Cu1	175.49 (5)
H11A—C11—H11B	107.8	C13—N1—C15	110.3 (3)
C11—C12—H12A	109.5	C13—N1—C11	112.4 (3)
C11—C12—H12B	109.5	C15—N1—C11	113.5 (3)
H12A—C12—H12B	109.5	C13—N1—H1	106.7
C11—C12—H12C	109.5	C15—N1—H1	106.7
H12A—C12—H12C	109.5	C11—N1—H1	106.7
H12B—C12—H12C	109.5	C1—O1—Cu1	121.79 (19)
N1—C13—C14	113.4 (3)	C1—O2—Cu2	123.1 (2)
N1—C13—H13A	108.9	C3—O3—Cu1	123.84 (19)
C14—C13—H13A	108.9	C3—O4—Cu2	120.88 (19)
N1—C13—H13B	108.9	C5—O5—Cu1	121.84 (19)
C14—C13—H13B	108.9	C5—O6—Cu2	123.3 (2)
H13A—C13—H13B	107.7	C7—O7—Cu2	121.1 (2)
C13—C14—H14A	109.5	C7—O8—Cu1	123.7 (2)
C13—C14—H14B	109.5	C9—O9—Cu2	138.64 (19)
H14A—C14—H14B	109.5	C16—O11—H11	109.5
			
Cu1^i^—O10—C9—O9	163.2 (2)	O8—Cu1—O3—C3	−7.4 (5)
Cu1^i^—O10—C9—C10	−17.2 (4)	O10^ii^—Cu1—O3—C3	169.8 (2)
N1—C15—C16—O11	−54.5 (4)	Cu2—Cu1—O3—C3	−9.9 (2)
O1—Cu1—Cu2—O7	97.97 (10)	O3—C3—O4—Cu2	3.4 (4)
O5—Cu1—Cu2—O7	−81.87 (10)	C4—C3—O4—Cu2	−178.2 (2)
O8—Cu1—Cu2—O7	8.74 (10)	O2—Cu2—O4—C3	−91.6 (2)
O3—Cu1—Cu2—O7	−171.90 (10)	O6—Cu2—O4—C3	74.9 (2)
O1—Cu1—Cu2—O4	−82.60 (9)	O9—Cu2—O4—C3	167.3 (2)
O5—Cu1—Cu2—O4	97.56 (10)	Cu1—Cu2—O4—C3	−8.2 (2)
O8—Cu1—Cu2—O4	−171.83 (10)	O6—C5—O5—Cu1	4.7 (4)
O3—Cu1—Cu2—O4	7.53 (9)	C6—C5—O5—Cu1	−175.0 (2)
O1—Cu1—Cu2—O2	7.13 (9)	O8—Cu1—O5—C5	−91.6 (2)
O5—Cu1—Cu2—O2	−172.71 (10)	O3—Cu1—O5—C5	73.3 (2)
O8—Cu1—Cu2—O2	−82.10 (10)	O10^ii^—Cu1—O5—C5	166.5 (2)
O3—Cu1—Cu2—O2	97.26 (10)	Cu2—Cu1—O5—C5	−9.3 (2)
O1—Cu1—Cu2—O6	−172.10 (10)	O5—C5—O6—Cu2	6.5 (4)
O5—Cu1—Cu2—O6	8.06 (9)	C6—C5—O6—Cu2	−173.8 (2)
O8—Cu1—Cu2—O6	98.67 (10)	O7—Cu2—O6—C5	75.7 (2)
O3—Cu1—Cu2—O6	−81.97 (10)	O4—Cu2—O6—C5	−96.2 (2)
C14—C13—N1—C15	62.7 (4)	O2—Cu2—O6—C5	−13.3 (6)
C14—C13—N1—C11	−65.1 (4)	O9—Cu2—O6—C5	170.1 (2)
C16—C15—N1—C13	163.9 (3)	Cu1—Cu2—O6—C5	−10.1 (2)
C16—C15—N1—C11	−68.9 (3)	O8—C7—O7—Cu2	7.2 (4)
C12—C11—N1—C13	−141.1 (3)	C8—C7—O7—Cu2	−173.0 (2)
C12—C11—N1—C15	92.8 (3)	O2—Cu2—O7—C7	72.4 (2)
O2—C1—O1—Cu1	6.5 (4)	O6—Cu2—O7—C7	−94.0 (2)
C2—C1—O1—Cu1	−171.9 (2)	O9—Cu2—O7—C7	173.6 (2)
O8—Cu1—O1—C1	73.2 (2)	Cu1—Cu2—O7—C7	−11.0 (2)
O3—Cu1—O1—C1	−91.8 (2)	O7—C7—O8—Cu1	4.9 (5)
O10^ii^—Cu1—O1—C1	174.9 (2)	C8—C7—O8—Cu1	−174.9 (2)
Cu2—Cu1—O1—C1	−9.2 (2)	O1—Cu1—O8—C7	−95.1 (3)
O1—C1—O2—Cu2	3.4 (4)	O5—Cu1—O8—C7	75.2 (3)
C2—C1—O2—Cu2	−178.2 (2)	O3—Cu1—O8—C7	−12.3 (6)
O7—Cu2—O2—C1	−93.5 (2)	O10^ii^—Cu1—O8—C7	170.5 (2)
O4—Cu2—O2—C1	78.2 (2)	Cu2—Cu1—O8—C7	−9.9 (2)
O6—Cu2—O2—C1	−4.6 (6)	O10—C9—O9—Cu2	−166.8 (2)
O9—Cu2—O2—C1	171.9 (2)	C10—C9—O9—Cu2	13.5 (5)
Cu1—Cu2—O2—C1	−7.8 (2)	O7—Cu2—O9—C9	−75.7 (3)
O4—C3—O3—Cu1	7.0 (4)	O4—Cu2—O9—C9	105.5 (3)
C4—C3—O3—Cu1	−171.4 (2)	O2—Cu2—O9—C9	15.5 (3)
O1—Cu1—O3—C3	75.3 (2)	O6—Cu2—O9—C9	−165.4 (3)
O5—Cu1—O3—C3	−94.9 (2)		

Symmetry codes: (i) *x*+1/2, −*y*+3/2, *z*; (ii) *x*−1/2, −*y*+3/2, *z*.

### Hydrogen-bond geometry (Å, °)

**Table:**

*D*—H···*A*	*D*—H	H···*A*	*D*···*A*	*D*—H···*A*
N1—H1···O10^ii^	0.93	1.97	2.832 (4)	153
N1—H1···O9^ii^	0.93	2.45	3.056 (3)	123
O11—H11···O9^ii^	0.84	2.04	2.840 (3)	159

Symmetry codes: (ii) *x*−1/2, −*y*+3/2, *z*.
